# Attention-deficit/hyperactivity disorder and smoking habits in pregnant women

**DOI:** 10.1371/journal.pone.0234561

**Published:** 2020-06-18

**Authors:** Anneli Andersson, Tor-Arne Hegvik, Qi Chen, Mina A. Rosenqvist, Liv Grimstvedt Kvalvik, Catarina Almqvist, Brian M. D’Onofrio, Catharina Hartman, Kari Klungsøyr, Jan Haavik, Catherine Tuvblad, Henrik Larsson

**Affiliations:** 1 School of Medical Sciences, Orebro University, Orebro, Sweden; 2 Department of Biomedicine, University of Bergen, Bergen, Norway; 3 Department of Medical Epidemiology and Biostatistics, Karolinska Institutet, Solna, Sweden; 4 Department of Global Public Health and Primary Care, University of Bergen, Bergen, Norway; 5 Pediatric Allergy and Pulmonology Unit at Astrid Lindgren Children’s Hospital, Karolinska University Hospital, Solna, Sweden; 6 The Department of Psychological and Brain Sciences at Indiana University, Bloomington, Indiana, United States of America; 7 Department of Psychiatry, University of Groningen University Medical Center, Groningen, The Netherlands; 8 Division for Mental and Physical Health, Norwegian Institute of Public Health, Bergen, Norway; 9 Division of Psychiatry, Haukeland University Hospital, Bergen, Norway; 10 School of Psychology, Law and Social Work, Orebro University, Orebro, Sweden; 11 Department of Psychology, University of Southern California, Los Angeles, California, United States of America; Chiba Daigaku, JAPAN

## Abstract

**Background:**

Attention-deficit/hyperactivity disorder (ADHD) has been associated with an increased risk of tobacco smoking, and more difficulties with smoking cessation compared to non-ADHD individuals. Women with ADHD may therefore show elevated rates of smoking during pregnancy.

**Aims:**

To examine the association between ADHD and smoking habits among pregnant women in Sweden and Norway.

**Methods:**

Women pregnant for the first time were identified in Sweden (n = 622,037), and Norway (n = 293,383), of which 1.2% (n = 7,444), and 1.7% (n = 4,951) were defined as having ADHD, respectively. Data on smoking habits were collected early and late in pregnancy.

**Results:**

In Sweden, ADHD was associated with an increased risk of smoking early in pregnancy, adjusted risk ratio (adjRR) 2.69 (95% confidence interval, 2.58–2.81), and late in pregnancy, adjRR 2.95 (2.80–3.10). Similar findings were observed in the Norwegian data, early in pregnancy, adjRR 2.31 (2.21–2.40), and late in pregnancy, adjRR 2.56 (2.42–2.70). Women with ADHD were more likely to continue smoking during pregnancy, compared to women without ADHD, both in Sweden adjRR 1.13 (1.10–1.17), and in Norway, adjRR 1.16 (1.12–1.20). Having a sibling diagnosed with ADHD was associated with an increased risk of smoking early and late in pregnancy, in both Sweden and Norway.

**Conclusions:**

Women with ADHD are considerably more likely to smoke early and late in (their first) pregnancy and are less likely to stop smoking between the two time points. Smoking, early and late in pregnancy, co-aggregates in families with ADHD. Smoking prevention and intervention programs should be targeted towards women with ADHD, specifically during their childbearing years, to ensure better mother and child outcomes.

## Introduction

Attention-Deficit/Hyperactivity Disorder (ADHD) is a common neurodevelopmental disorder with a strong genetic component [1], that often debuts during childhood and may persist into adulthood [2,3]. Several health-risk behaviors are common among individuals with ADHD [4], including smoking [5]. Adolescents and adults diagnosed with ADHD are about twice as likely to smoke compared to individuals without ADHD [6,7]. Individuals diagnosed with ADHD are also more likely to report earlier smoking initiation and become daily smokers compared to individuals without ADHD [8]. However, some of these studies are based on relatively small and non-representative selected samples thus the knowledge of the burden of smoking among individuals diagnosed with ADHD is still limited.

Smoking during pregnancy is considered a serious, avoidable risk behavior that is believed to increase the likelihood of adverse pregnancy related outcomes in both the mother and the child. For example, maternal smoking during pregnancy increases the risk of low birth weight [9,10], and preterm birth [11], results that also have been validated in genetically sensitive designs [12]. Maternal smoking during pregnancy has also been associated with an increased risk of stillbirth [13] and infant mortality [14]. It is further estimated that 6% of global female deaths are due to smoking [15]. A recent study reported more nicotine dependence and higher cigarette consumption in women compared to males diagnosed with ADHD [16]. It has also been demonstrated that individuals with ADHD experience more difficulties with smoking cessation compared to individuals without ADHD [8]. Women with ADHD may therefore show elevated rates of smoking during pregnancy.

Like ADHD [17], smoking habits [18] also tend to aggregate within families, i.e., one individual’s smoking increases the risk of his/her family members to also engage in smoking. However, whether ADHD and smoking co-aggregates is currently not known. A large molecular genetic study demonstrated positive genetic correlations between ADHD and smoking habits [19], and ADHD polygenic scores have been found to be associated with more smoking during pregnancy [20]. However, these findings need to be replicated in other settings (e.g., pregnant women), using other study designs (e.g., familial co-aggregation study).

To attain more knowledge regarding ADHD and smoking during pregnancy, we used population-based register data from Sweden and Norway to examine the associations between ADHD and smoking during pregnancy, using a cohort design. We addressed the following three research questions:

1. Are women diagnosed with ADHD more likely to smoke during their first pregnancy compared to women without ADHD?
2. Are women diagnosed with ADHD more likely to continue smoking during pregnancy, compared to women without ADHD?
3. Do ADHD and smoking during pregnancy co-aggregate in families?

## Methods

### Study population Sweden

The Medical Birth Register of Sweden (MBRS) was established in 1973 with the purpose of collecting health data on all Swedish pregnancies [21]. In Sweden, as in the other Nordic countries, each individual residing in the country has a unique personal identification number (PIN) that permits linkage between public registers and databases.

Based on compulsory notification, the MBRS includes information on all live births and stillbirths (from 28 gestational weeks until 2008, and from 22 gestational weeks thereafter). We used the MBRS to identify all unique singleton pregnancies between 2000 and 2013 (n = 856,096). We excluded women with missing information on the PIN, birth year, or parity (n = 7). We further restricted the sample to women who were pregnant for the first time, resulting in 622,037 pregnancies (of which n = 2,277 (0.4%) ended in stillbirth). Next we used the Swedish Multi-Generation Register [22] to link the index women to their full siblings. We only included first pregnancies to avoid correlated data, i.e., same mother contributing with data more than once. We further only included singletons due to potential differences on follow-up in multiparous pregnancies.

This study was approved by the Regional Ethical Review board in Stockholm, Sweden (DNR: 2013/862-31/5).

### Study population Norway

The Medical Birth Registry of Norway (MBRN) is a mandatory population-based register established in 1967 to record information on all pregnancies in Norway from 16^th^ gestational week, and from the 12^th^ gestational week from 2002 [23]. Maternal smoking habits have been available in the registry since December 1998. We used the MBRN to identify all unique singleton pregnancies between 1999 and 2012 (n = 488,745). We excluded women with missing information on PIN, birth year, or parity (n = 7,583), and those that had died or emigrated prior to 2004 (n = 1,998) which was the year that the Norwegian Prescription Database (NorPD) was established. We restricted the sample to only include women who were pregnant for the first time leaving us with a total of 293,383 pregnancies (of which n = 1,599 (0.5%) ended in stillbirth). The MBRN was further used to identify the siblings of the pregnant women.

The Western Norway Regional Ethics Committee has approved the use of the Norwegian data for this study (2011/2272).

### Smoking Sweden

The MBRS has collected information on maternal smoking at the first antenatal visit (usually during the first trimester) since 1983, and smoking habits in week 30–32 since 1991. Due to a high proportion of missing smoking information in week 30–32, data for this point in pregnancy has only been available for research since 2000 [21]. Smoking habits are collected by midwives, using self-reports, and registered as: “not smoke”, “1–9 cigarettes/day” or “more than 9 cigarettes/day”. We dichotomized smoking habits by defining “not smoke” as “non-smoking” whereas “1–9 cigarettes/day” or “more than 9 cigarettes/day” was defined as “smoking”. Smoking at the first antenatal visit and in week 30–32 will be referred to as smoking early in pregnancy and late in pregnancy.

### Smoking Norway

Since December 1998, the MBRN has collected information on smoking at the first antenatal visit (in the first trimester, gestational week 0–12) and in the last trimester (from gestational week 29). Information on smoking is obtained by a physician or a midwife during antenatal care where a pregnant woman is defined as a “non-smoker”, “occasional, but not daily smoker” or “daily smoker”. We defined smoking in the first and last trimester as early versus late in pregnancy. To harmonize the Swedish and Norwegian data, the smoking variables “non-smoker” and “occasional, but not daily smoker” were defined as “non-smoking” while “daily smoker” was defined as “smoking”.

### ADHD Sweden

The Swedish National Patient Register (SNPR) [24] provides complete information on all psychiatric inpatient care since 1987, and outpatient care from 2001. The Swedish Prescribed Drug Register (SPDR) was established in 2005 and contains data on dispensed medication [25]. Women (and their full siblings) were defined as having ADHD if they had ever received a diagnosis of ADHD in the SNPR (International Classification of Disease (ICD-9: 314 or ICD-10: F90) or ever been dispensed a drug (SPDR) used almost exclusively in the treatment of ADHD (Anatomical Therapeutic Chemical (ATC) codes: methylphenidate (N06BA04), amphetamine (N06BA01), dexamphetamine (N06BA02), atomoxetine (N06BA09), or lisdexamphetamine (NO6BA12)). This resulted in a total of 7,444 (1.2%) women being defined as having a diagnosis of ADHD. We used SNPR data from 1987 to 2013 and SPDR data from 2005 to 2013.

### ADHD Norway

The Norwegian Patient Registry (NNPR) was established in 1997 but has only included unique PINs for registered patients since 2008 and can therefore only be linked with other registers since that year. It covers inpatient and outpatient specialist somatic and psychiatric health care in Norway [26]. The Norwegian Prescription Database (NorPD) was established in 2004 and collects information on dispensed drug prescriptions from all Norwegian pharmacies including the indication for reimbursed medication, partly since 2004 and completely since 2008 [27]. Women were defined as having ADHD if they had ever received a diagnosis of ADHD (ICD-10: F90) in the NNPR or had ever been dispensed an ADHD-specific drug (methylphenidate (N06BA04), amphetamine (N06BA01), dexamphetamine (N06BA02), atomoxetine (N06BA09), or lisdexamphetamine (NO6BA12)). For the years 2008–2016 we also required that the indication for drug prescription was ICD-10: F90 or ICPC: P81. In total, 4,951 (1.7%) of the women were defined as having ADHD. We used NNPR data from 2008 to 2016 and NorPD data from 2004 to 2016. As in Sweden, linkage between the registers was based on anonymized PINs.

### Covariates in Sweden and Norway

Information on the *year of childbirth* was retrieved from the MBRS (2000–2013) and the MBRN (1999–2012) in order to adjust for potential period effects since both smoking prevalence and diagnostic practises may have changed over time.

Socioeconomic Status (SES) has been shown to be associated with both smoking [28] and ADHD [29] with lower SES being associated with higher smoking rates [30], and smoking cessation less likely to be successful [31]. SES could therefore act as a confounder (or a mediator) in the association between ADHD and smoking. We used data from the Longitudinal Integration Database for Health Insurance and Labour Market (LISA) [32], and the National education database of Norway to define a proxy measure of SES as the highest recorded level of education achieved by the mothers of the primiparous women. This variable was categorized as: <9 years: 1; 9 years: 2; 10–11 years: 3; 12 years: 4; 13–14 years: 5; 15 years: 6; >15 years: 7 and will be referred to as *maternal education*.

ADHD is highly comorbid with other psychiatric conditions [33,3], therefore we defined women to have been diagnosed with *any comorbid psychiatric disorder* if they had been diagnosed with any non-ADHD/non-tobacco-addiction psychiatric disorder in the SNPR or in the NNPR (see Table 1).

**Table 1:** ICD codes included in the combined covariate any psychiatric disorder.

Disorders	
**ICD-9 codes**	'291' '292' '293' '294' '295' '296' '297' '298' '299' '300' '301' '302' '303' '304' '306' '307' '308' '309' '310' '311' '312' '313' '315' '316' '317' '318' '319' '305A' '305X'
**ICD-10 codes**	'F2' 'F3' 'F4' 'F5' 'F6' 'F7' 'F8' 'F10' 'F11' 'F12' 'F13' 'F14' 'F15' 'F16' 'F18' 'F19' 'F91' 'F92' 'F93' 'F94' 'F95' 'F98' 'F99'

### Statistical analyses

Data management and descriptive analyses were performed with SAS software version 9.4 (SAS Institute Inc., Cary, NC), R and R Studio. We used logistic regression and regression standardization with the stdReg-package to estimate risk ratios (RRs) and 95% confidence intervals. In the logistic regression models, ADHD was the predictor and smoking early and late in pregnancy were the outcomes. Unadjusted estimates may be more appropriate for clinical prediction, while adjusted estimates might be more informative under a causal framework. Therefore, both unadjusted and adjusted estimates will be presented.

1. First, we estimated the prevalence and association between ADHD and smoking in pregnant women with versus without ADHD. Second, we adjusted for maternal education and year of childbirth. Third, we adjusted for psychiatric comorbidities.
2. We defined women who reported that they smoked at two consecutive measurements (i.e., *early in pregnancy* and *late in pregnancy*) as continued smokers. Women with only one measurement of smoking during pregnancy were excluded. Thus, we could compare the rate of continuous smoking through the pregnancy in women diagnosed with versus without ADHD, while adjusting for maternal education, year of childbirth and psychiatric comorbidities.
3. We assessed the familial co-aggregation of ADHD and smoking during pregnancy, i.e., whether there was an association between smoking during pregnancy and having a full sibling with ADHD, while adjusting for period effects and ADHD in the index women (which in principle corresponds to excluding index women with ADHD). We further examined the absolute risk and risk difference of smoking during pregnancy in those women with versus without a sibling diagnosed with ADHD to help guide clinical prediction and decision making.

## Results

The demographic properties of the Swedish and the Norwegian cohorts are presented in Table 2.

**Table 2:** Study characteristics for Sweden and Norway.

	Sweden		Norway	
Variables	ADHD (%)	Non-ADHD (%)	ADHD (%)	Non-ADHD (%)
**Missing Smoking**				
Early in pregnancy	417 (5.6)	31,510 (5.1)	667 (13.5)	50,107 (17.4)
Late in pregnancy	1,122 (15.1)	49, 603 (8.1)	857 (17.3)	59,917 (20.8)
**Maternal education, years**				
<9 years	413 (5.8)	50,520 (9.7)	6 (0.1)	773 (0.4)
9 years	1,205 (17.1)	61,272 (11.7)	1,689 (38.0)	57,706 (26.0)
10–11 years	2,983 (42.3)	200,153 (38.3)	1,001 (22.5)	70,869 (31.9)
12 years	872 (12.3)	55,961 (10.7)	852 (19.2)	36,028 (16.2)
13–14 years	725 (10.3)	66,619 (12.7)	95 (2.1)	4,914 (2.2)
15 years	830 (11.8)	84,980 (16.3)	741 (16.7)	46,680 (21.0)
>15 years	30 (0.4)	2,899 (0.6)	59 (1.3)	5,281 (2.4)
Psychiatric Comorbidity*				
Depression	3,537 (47.5)	35,184 (5.7)	1,520 (30.1)	20,167 (7.0)
Substance use disorder	2,219 (29.8)	16,013 (2.6)	853 (17.2)	3,976 (1.4)
Bipolar disorder	1,167 (15.7)	4,424 (0.7)	396 (8.0)	2,451 (0.8)
Personality disorders	1,563 (21.0)	5,912 (9.6)	628 (12.7)	3,243 (1.1)
Schizophrenia	259 (3.5)	2,346 (0.4)	90 (1.8)	1,034 (0.4)
Any psychiatric disorder*	6,296 (84.6)	88,134 (14.3)	3,311 (67.0)	50,114 (17.4)

*Sum is more than 100% as some women will have more than 1 additional disorder.

### Are women diagnosed with ADHD more likely to smoke during their first pregnancy compared to women without ADHD?

In Sweden, a total of 33.0% of the women with ADHD smoked early in pregnancy, compared to 7.3% in those without ADHD, equivalent to an unadjusted risk ratio (RR) of 4.55 (4.40–4.71). Moreover, 25.9% of the women with ADHD smoked late in pregnancy compared with 4.9% of the women without ADHD, RR = 5.33 (5.10–5.56). Similar results were noted in Norway where 44.5% of the women with ADHD smoked early in pregnancy, compared to 15.0% in those without ADHD, equivalent to an unadjusted RR of 2.96 (2.86–3.06). Moreover, 31.1% of women with ADHD reported that they smoked late in pregnancy compared with 8.9% of the women without ADHD, RR = 3.48 (3.31–3.64) (Table 3).

**Table 3:** Association between ADHD and smoking during pregnancy in women diagnosed with ADHD compared to women without ADHD, in Sweden and Norway.

			Prevalence of smoking (%)		Unadjusted RRs	Adjusted RRs*	Adjusted RRs**	
	*Total Non-ADHD*	*Total ADHD*	Non-ADHD	ADHD	RR (95% CI)	RR (95% CI)	RR (95% CI)	P value
**Sweden**								
Smoking early in pregnancy	583,083	7,027	42,303 (7.3)	2,322 (33)	4.55 (4.40–4.71)	4.40 (4.25–4.55)	**2.69 (2.58–2.81)**	<0.001
Smoking late in pregnancy	564,990	6,322	27,498 (4.9)	1,639 (25.9)	5.33 (5.10–5.56)	5.17 (4.94–5.39)	**2.95 (2.80–3.10)**	<0.001
**Norway**								
Smoking early in pregnancy	238,325	4,284	35,836 (15.0)	1,907 (44.5)	2.96 (2.86–3.06)	2.72 (2.62–2.82)	**2.31 (2.21–2.40)**	<0.001
Smoking late in pregnancy	228,515	4,094	20,441 (8.9)	1,273 (31.1)	3.48 (3.31–3.64)	3.21 (3.06–3.37)	**2.56 (2.42–2.70)**	<0.001

*Adjusted for year of childbirth, and maternal education

**Adjusted for year of childbirth, maternal education, and psychiatric comorbidity.

In Sweden, after adjustment for maternal education, and year of childbirth, women with ADHD were still significantly more likely to smoke early in pregnancy adjRR = 4.40, (4.25–4.55), and late in pregnancy adjRR = 5.17, (4.94–5.39), compared to women without ADHD. Similar results were found in Norway, where women with ADHD were significantly more likely to smoke early in pregnancy adjRR = 2.72 (2.62–2.82), and late in pregnancy adjRR = 3.21 (3.06–3.37), compared to women without ADHD. Further adjustment for any other psychiatric disorders attenuated these associations, but the results remained robust in both Sweden [early in pregnancy adjRR = 2.69 (2.58–2.81), and late in pregnancy adjRR = 2.95 (2.80–3.10)], and Norway [early in pregnancy adjRR = 2.31 (2.21–2.40) and late in pregnancy adjRR = 2.56 (2.42–2.70)] (Table 3).

### Are women diagnosed with ADHD more likely to continue smoking during pregnancy, compared to women without ADHD?

In Sweden, women with ADHD had an increased risk of continued smoking during pregnancy (e.g. reported smoking both early in pregnancy and late in pregnancy) adjRR = 1.19 (1.16–1.22), compared to women without ADHD. Similar results were found in Norway adjRR = 1.20 (1.16–1.24). With further adjustment for any other psychiatric disorders in both countries, the associations were somewhat attenuated but were still present (Table 4).

**Table 4:** Association between ADHD and continued smoking in women with ADHD compared to women without ADHD, in Sweden and Norway. Women with only one measurement of smoking during pregnancy are excluded.

			Prevalence of continued smoking (%)		Unadjusted RRs	Adjusted RRs*	Adjusted RRs**	
	*Total Non-ADHD*	*Total ADHD*	Non-ADHD	ADHD	RR (95% CI)	RR (95% CI)	RR (95% CI)	P value
**Sweden**								
Smoking early *and* late in pregnancy	31,672	1,850	20,592 (65.0)	1,418 (76.6)	1.18 (1.15–1.21)	1.19 (1.16–1.22)	**1.13 (1.10–1.17)**	<0.001
**Norway**								
Smoking early *and* late in pregnancy	29,437	1,669	17,066 (58.0)	1,157 (69.3)	1.20 (1.16–1.24)	1.20 (1.16–1.24)	**1.16 (1.12–1.20)**	<0.001

*Adjusted for year of childbirth, and maternal education

**Adjusted for year of childbirth, maternal education, and psychiatric comorbidity.

### Do ADHD and smoking during pregnancy co-aggregate in families?

In Sweden, women who had a sibling diagnosed with ADHD were more likely to smoke both early (adjRR = 2.16, 2.07–2.25) and late in pregnancy (adjRR = 2.36, 2.24–2.49), compared to women without a sibling diagnosed with ADHD, after adjusting for ADHD in the index women. The absolute risk of smoking early in pregnancy in women with a sibling diagnosed with ADHD was 18.0%, with a risk difference of 10.4%-points compared to women without a sibling with ADHD. Corresponding absolute risk of smoking late in pregnancy was 13.8%, with a risk difference of 8.6%-points.

Similar results were found in Norway. Women who had a sibling diagnosed with ADHD were more likely to smoke early in pregnancy (adjRR = 1.61, 1.53–1.69) and late in pregnancy (adjRR = 1.80, 1.69–1.92), compared to women without a sibling diagnosed with ADHD, after adjusting for ADHD in the index women. The absolute risk of smoking early in pregnancy in women with a sibling diagnosed with ADHD was 26.0%, with a risk difference of 10.8%-points compared to women without a sibling with ADHD. Corresponding absolute risk late in pregnancy was 17.5%, with a risk difference of 8.5%-points (Table 5).

**Table 5:** Association between having a sibling diagnosed with ADHD and smoking during pregnancy in women, in Sweden and Norway.

	Unique siblings		Adjusted*		Absolute Risk %		Risk Difference %
	*With ADHD*	*Without ADHD*	RR (95% CI)	P value	Non-ADHD Sibling	ADHD Sibling	
**Sweden**							
Smoking early in pregnancy	418,536	11,807	**2.16 (2.07–2.25)**	<0.001	7.6 (7.5–7.7)	18.0 (17.3–18.8)	10.4 (9.7–11.2)
Smoking late in pregnancy	343,466	9,881	**2.36 (2.24–2.49)**	<0.001	5.2 (5.1–5.3)	13.8 (13.1–14.5)	8.6 (8.0–9.3)
**Norway**							
Smoking early in pregnancy	232,705	5,679	**1.61 (1.53–1.69)**	<0.001	15.2 (15.0–15.4)	26.0 (24.8–27.2)	10.8 (9.6–12.0)
Smoking late in pregnancy	224,111	5,462	**1.80 (1.69–1.92)**	<0.001	8.9 (8.8–9.1)	17.5 (16.4–18.5)	8.5 (7.5–9.6)

*Adjusted for year of childbirth, and ADHD in index women.

## Discussion

In this cross-nation population-based study, we demonstrate that a diagnosis of ADHD was strongly associated with smoking during pregnancy in both Sweden and Norway, even after adjusting for maternal education, year of childbirth and the presence of comorbid psychiatric disorders. In comparison to pregnant women without ADHD, pregnant women diagnosed with ADHD were also more likely to report smoking both early and late in pregnancy. Our study further demonstrated that smoking both early and late in pregnancy co-aggregates in families with ADHD. These findings show that among women, ADHD is a strong and clinically relevant predictor of smoking during pregnancy.

Our first finding that women with ADHD were considerably more likely to smoke in their first pregnancy compared to women without ADHD, is in line with previous research on non-pregnant study populations [8]. Women with ADHD are more likely to become pregnant during their teenage years [34] and have an earlier smoking initiation [8] indicating that adolescent girls and young women with ADHD may be a vulnerable group in need of support. Thus, our finding highlights the importance of early intervention and counseling by health care providers to help prevent the development of regular and established smoking habits in women diagnosed with ADHD. Smoking cessation would benefit both immediate and long-term outcomes in the mother and child.

Our second main finding indicates that women diagnosed with ADHD are more likely to continue smoking throughout pregnancy compared to women without ADHD as they were more likely to report smoking at the two consecutive time-points (early and late in pregnancy). Previous research has suggested that these differences in smoking cessation may be the result of greater withdrawal severity in individuals with ADHD compared to those without ADHD [6]. Smoking cessation early in pregnancy reduce the risk of stillbirth and prematurity to the level of non-smokers [35]. This highlights the need for better smoking cessation interventions for pregnant women with ADHD who smoke.

Our third finding demonstrated that smoking during pregnancy and ADHD co-aggregate in families. Having a sibling with ADHD increases the risk of smoking during pregnancy in women that do not have ADHD themselves, indicating a shared familial liability to both ADHD and smoking during pregnancy. This is in line with recent findings of genetic correlations between ADHD and several smoking-related traits, including lung cancer [19] as well as a study that reported an association between an ADHD polygenic score and smoking during pregnancy [20]. Of clinical interest, the familial co-aggregation of ADHD and smoking demonstrates that a family history of ADHD may be used by health care providers to identify women that might be in need for additional support and help with smoking cessation. Our findings may also generalize to outside of pregnancy situations. Therefore, our findings suggest that a family history of ADHD could be used to identify children and adolescents that are at risk of starting smoking.

It is important to highlight that there are several potential mechanisms underlying the association seen between ADHD and smoking while pregnant. Both ADHD and smoking are known to be highly heritable [36,37]. Previous research has also found genetic markers associated with both ADHD and smoking [38,39,40,41]. This suggests that neurobiological factors that contribute to ADHD symptoms also contributes to an individual’s risk to commence and continue smoking [42]. Behavioral risk factors, such as deficient impulse-control could in addition to genes potentially explain why individuals diagnosed with ADHD are more prone to engage in adverse health behaviors, such as smoking and other types of substance abuse. Further, several social influences (e.g., smoking habits in family and friends) could also increase the risk for smoking in ADHD individuals [43].

### Strengths and limitations

By using population-based registries with compulsory notification, our study is not limited by restrictions in statistical power, and unlikely to suffer from selection bias. The results in the present study shows that the association between ADHD and smoking during pregnancy is robust across Nordic nations. It is, however, important to note that a higher proportion of women with and without ADHD smoke in Norway compared to Sweden (see S1 and S2 Figs). The higher smoking prevalence in Norway likely reflects true differences across countries, and between registries. For example, smoking during pregnancy has been higher in Norway compared to Sweden in previous years [44] Yet, it seems as if smoking during pregnancy has reduced more in Norway compared to Sweden [44]. In addition to the time periods not completely matching (as we sought to maximize the statistical power in our study given the data available to us), women in Norway can refrain from having their smoking data registered, leading to a higher number of missing values on the smoking variables compared to Sweden.

Another strength includes the large datasets and the possibilities to adjust for other psychiatric disorders in an effort to investigate whether it is ADHD itself, or common psychiatric comorbidities, that “drive” the association between ADHD and smoking during pregnancy. However, there could be limitations in these assumptions as other traits, including somatic diseases, may impact both ADHD severity and adverse health behaviors. Yet, these psychiatric disorders frequently co-occur with ADHD and seemed appropriate based on previous studies [45].

Another strength of the study is our definition of ADHD. This definition has previously been shown to have a high correlation with ADHD symptoms (derived from the Swedish twin register) [46]. However, it is important to mention that the use of clinical diagnoses of ADHD in the Swedish and Norwegian registers likely captures the more burdened ADHD cases.

The registers also enabled us to conduct sensitivity analyses to exclude some alternative explanations. For some women, the definition of the predictor will be after the definition of the outcome. However, as ADHD is considered being a neurodevelopmental disorder present from young age (1,2) it is likely that the predictor was present before the outcome of interest (smoking early and late in pregnancy). Sensitivity analyses restricted to first pregnancies between 2007 and 2013, where the ADHD definition only included women who were defined as having ADHD prior to becoming pregnant, revealed largely unchanged results (results not presented). This indicates that bias from period effects as well as the risk of reverse causation, are unlikely to influence the associations seen between ADHD and smoking during pregnancy. Additionally, we were able to demonstrate that factors surrounding artificial fertilization (e.g., smoking cessation may be mandatory) did not substantially alter our results. We were also able to demonstrate that ADHD-medication during pregnancy did not introduce a substantial amount of bias to our estimates (results for these two separate analyses are not shown).

In this study, we treated women who reported no daily smoking as non-smokers, which could lead to some misclassification. Consequently, women who smoke occasionally, but not on a daily basis, were included in the non-smoker category. However, sensitivity analysis from the Norwegian data, where both “daily smokers” and “occasional, but not daily smoker” were treated as smokers lead to largely similar results as the main analyses (results not presented), which further supports our findings. Further, the stigma surrounding smoking during pregnancy might lead to underreporting. However, high agreement between the smoking information in the MBRS and maternal serum cotinine has previously been reported [47].

Finally, by investigating smoking late in pregnancy, we could demonstrate that women diagnosed with ADHD were more likely to continue smoking throughout pregnancy, compared to women without ADHD. In the present study, we assumed that women who smoked at the two consecutive time points represented women that continued to smoke through the whole time-period. Importantly, we did not have information about periods without smoking in-between the time-points. However, we believe it is unlikely that more detailed information would have led to a conclusion different from the one presented in our study, namely that among pregnant women who smoke in the beginning of pregnancy, those with ADHD have a higher risk of being daily smokers also late in pregnancy.

## Conclusions

To conclude, our large cross-nation population-based study indicates that women with ADHD are more likely to smoke both in the beginning and at the end of the pregnancy and have a lower likelihood of smoking cessation during pregnancy, compared to women without ADHD. Having a sibling with ADHD is associated with an increased likelihood of smoking during pregnancy suggesting a shared familial liability. The considerably increased risk of smoking throughout pregnancy among women with ADHD highlights the importance of early interventions and professional support to this group, especially as smoking avoidance and/or cessation would ensure better mother and child-outcomes.

## Supporting information

S1 Fig Proportion of smoking early in pregnancy among those with versus without ADHD, in Sweden. (DOCX)

S2 Fig Proportion of smoking early in pregnancy among those with versus without ADHD, in Norway. (DOCX)
