# Supplementary material for: Attention-deficit/hyperactivity disorder and smoking habits in pregnant women
Source: PLoS One. 2020 Jun 18;15(6):e0234561. doi: 10.1371/journal.pone.0234561 (PMC7302708; doi:10.1371/journal.pone.0234561)

**Figure S2.** Proportion of smoking early in pregnancy among those with versus without ADHD, in Norway.


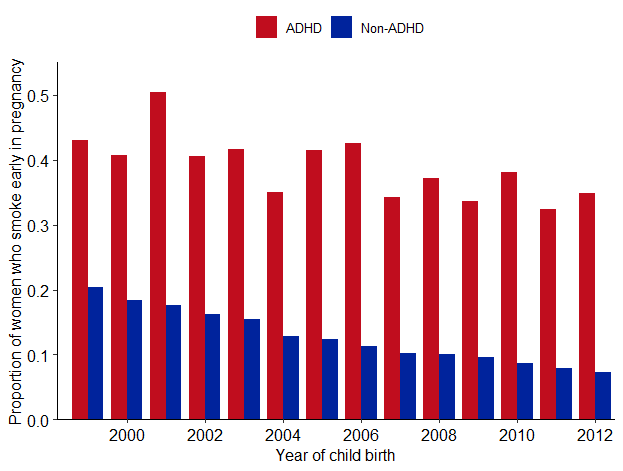

Supplement: S2 Fig — (DOCX) [file pone.0234561.s002.docx]
